# Mutation of the CH1 Domain in the Histone Acetyltransferase CREBBP Results in Autism-Relevant Behaviors in Mice

**DOI:** 10.1371/journal.pone.0146366

**Published:** 2016-01-05

**Authors:** Fei Zheng, Lawryn H. Kasper, David C. Bedford, Stephanie Lerach, Brett J. W. Teubner, Paul K. Brindle

**Affiliations:** 1 Department of Biochemistry, St Jude Children’s Research Hospital, Memphis, TN 38105, United States of America; 2 Department of Developmental Neurobiology, St Jude Children’s Research Hospital, Memphis, TN 38105, United States of America; University of Insubria, ITALY

## Abstract

Autism spectrum disorders (ASDs) are a group of neurodevelopmental afflictions characterized by repetitive behaviors, deficits in social interaction, and impaired communication skills. For most ASD patients, the underlying causes are unknown. Genetic mutations have been identified in about 25 percent of ASD cases, including mutations in epigenetic regulators, suggesting that dysregulated chromatin or DNA function is a critical component of ASD. Mutations in the histone acetyltransferase CREB binding protein (CBP, CREBBP) cause Rubinstein-Taybi Syndrome (RTS), a developmental disorder that includes ASD-like symptoms. Recently, genomic studies involving large numbers of ASD patient families have theoretically modeled CBP and its paralog p300 (EP300) as critical hubs in ASD-associated protein and gene interaction networks, and have identified *de novo* missense mutations in highly conserved residues of the CBP acetyltransferase and CH1 domains. Here we provide animal model evidence that supports this notion that CBP and its CH1 domain are relevant to autism. We show that mice with a deletion mutation in the CBP CH1 (TAZ1) domain (*CBP*^Δ*CH1/*Δ*CH1*^) have an RTS-like phenotype that includes ASD-relevant repetitive behaviors, hyperactivity, social interaction deficits, motor dysfunction, impaired recognition memory, and abnormal synaptic plasticity. Our results therefore indicate that loss of CBP CH1 domain function contributes to RTS, and possibly ASD, and that this domain plays an essential role in normal motor function, cognition and social behavior. Although the key physiological functions affected by ASD-associated mutation of epigenetic regulators have been enigmatic, our findings are consistent with theoretical models involving CBP and p300 in ASD, and with a causative role for recently described ASD-associated CBP mutations.

## Introduction

Autism spectrum disorders (ASDs) are distinguished by repetitive behaviors, deficits in social interaction, and impaired communication skills [1–4]. The genetics of these disorders are often complex and the cause of ASD is unknown for many patients. Single-gene syndromes account for only 7–9% of ASDs with heterogeneneous comorbidities including hyperactivity, intellectual disability, and other neurological symptoms [4,5]. Many of the ASD associated mutations that have been found occur in genes that encode epigenetic and chromatin regulators, suggesting that aberrant chromatin or DNA function contributes to ASD [6]. Although more than 667 ASD candidate genes have been defined so far (Source: SFARI Gene 2.0, [7]), only a limited number of ASD mouse models have been developed. Therefore, mouse models with mutations in ASD syndromic genes are valuable for studying the converging mechanisms for ASDs that arise from mutations in different genes with biologically related roles.

CBP (CREBBP, CREB binding protein) and its paralog p300 (EP300) comprise the KAT3 family of histone acetyltransferases (HATs) [8], and mainly function as transcriptional co-activators [9]. CBP has one histone acetyltransferase domain (HAT domain) and several protein-binding domains including KIX, CH1 and CH3, the latter of which are principally modeled to recruit CBP to DNA-bound transcription factors (S1 Fig). We have previously described knock-in mice having an in-frame 52 amino acid deletion within the highly conserved 88 residue CBP CH1 domain [10]. This deletion removes amino acids 342–393, which includes the first two of four alpha helices in the CH1 domain and five of its twelve zinc-chelating residues, thereby disrupting the domain structure and ability to bind transcriptional regulators (e.g. HIF and CITED2) without affecting CBP expression level or acetyltransferase activity [10–13].

Heterozygous mutations in *CREBBP* and, to a lesser extent, *EP300* cause Rubinstein-Taybi Syndrome (RTS), a congenital condition mainly characterized by mental retardation, distinctive facial features, and broad toes and thumbs [14,15]. Mice with heterozygous CBP null or truncating mutations (and described here, a CH1 domain mutation) have craniofacial anomalies and memory deficits, and are models of RTS (S1 Table) [16–19]. However, none of these models have been reported to present autism-relevant behaviors.

RTS is only peripherally defined as an ASD because not all patients exhibit ASD-relevant symptoms such as impaired motor skills, stereotyped hand movements, and sociability deficits [20–23]. Nevertheless, *CREBBP* is considered an ASD correlated gene in humans and is listed in autism gene databases [24,25]. Supporting this notion, recent exome sequencing of thousands of ASD patient families has led to both CBP and p300 being modeled as central components (i.e. “hubs”) of a theoretical network of genes and proteins disrupted in ASD [6]. Another recent study [26] provides additional support for such theoretical models, where Iossifov *et al*. identified seven *de novo CREBBP* and *EP300* mutations in ASD patients (S2 Table). Two of these mutations are silent, but five are missense mutations, including three that are in the histone acetyltransferase enzymatic domain, and one in the CBP CH1 domain (which is the focus of our study). CBP and p300 are large proteins (>2400 aa), which makes it especially intriguing that these ASD mutations occur in two critical functional domains. Moreover, the mutated CBP and p300 residues identified in ASD are highly conserved and for three of the mutations, including the one in the CH1 domain, the residues are absolutely conserved in all taxa with CBP/p300 represented in the NCBI database, including insects, worms, and sponges.

To determine whether mutation of the CH1 domain leads to autism-relevant phenotypes, we examined the behavior and hippocampal synaptic plasticity of *CBP*^Δ*CH1/*Δ*CH1*^ mice and found similarities to many of the phenotypes reported for ASD-relevant mouse models.

## Materials and Methods

### Animals

Generation of *CBP*^Δ*CH1/*Δ*CH1*^ mice has been described previously [10]. All experimental animals were C57BL/6 X 129Sv F1 hybrid mice, generated from congenic heterozygous parents backcrossed more than 20 times. The heterozygous parental mouse lines (stock numbers 25531 and 25172) are available from JAX (Bar Harbor, ME, USA). All experiments followed protocols approved by the Institutional Animal Care and Use Committee of St. Jude.

### MicroCT Scan

MicroCT scan was performed to assess craniofacial defects. The data were acquired on a dedicated *ex vivo* microCT Scanner (LocusSP Specimen CT, GE Healthcare) at 28 μm isotropic voxel size with 720 projections, an integration time of 1,700 ms, photon energy of 80 keV and current of 70 μA. Data processing was performed using MicroView (GE Healthcare) and are presented as rendered isosurfaces.

### Behavior

All behavioral tests were performed on adult male *CBP*^Δ*CH1/*Δ*CH1*^ mice and their heterozygous and wild type littermates (2–6 month old unless mentioned otherwise). The experimenters were blind to the mouse genotypes during the tests. In total, five cohorts of mice were used for all of the behavioral tests. When several behavioral tests were performed on the same cohorts of mice, the order was open field test, elevated plus maze test, repetitive forelimb movement assay, recognition memory, wire hang assay, grip-strength assay, self-grooming assay, nest-building assay, three-chamber assay, rotarod assay, resident-intruder assay and hot plate assay. The mice were allowed to rest at least one week before social behavioral tests, cognition tests, and rotarod test, and at least two days before all other tests. The mice were handled daily for at least 5 days prior to performing the first behavior test. They were also allowed to habituate for 30 minutes in the test room prior to each test.

#### Repetitive forelimb movement assay

In this assay to test for repetitive behavior, mice were suspended by their tails for 15 seconds, and their forelimb movements were observed and recorded using the following scale: 0 (no repetitive movements), 1 (occasional repetitive movements), or 2 (continuous repetitive movements). Two independent assays were carried out and the average of the scores was used.

#### Self-grooming assay

This assay was used to test a common repetitive behavior that is often prolonged in mouse models of autism [2]. Mice were singly transferred to a fresh cage and left for a 10-minute adaptation period. In the following 10 minutes, the amount of time spent self-grooming was recorded. The experiments were performed under ambient light at about 200 Lux without background noise, as previously described [27].

#### Hot plate assay

A hot plate (SD instruments) at 55±0.1°C was used to assay the nociception response of the mice. Mice were gently placed on the plate and the latency until they showed jumping, squealing, or licking of the hind paws was recorded.

#### Open field test

To assess general locomotor and exploratory activities, an open-field photo-beam recording system (SD Instrument) was used to record the activity of the mouse in a novel clear Plexiglas box (40 cm × 40 cm) for 30 minutes with a background white noise of 60 dB. The mouse’s travel distance, travel speed, and rearing were recorded and quantified by the manufacturer’s software.

#### Elevated plus maze test

In this test to assess anxiety, an elevated maze (San Diego Instrument) standing 40 cm above the floor with two open arms and two closed arms (enclosed by walls but no ceiling, all arms are 30 cm long and 5 cm wide, the walls are 15 cm high) arranged in a cross or plus shape was used. The mouse being tested was placed alone at the center of the maze, facing one of the open arms. The number of entries the mouse made into the open and closed arms, as well as the duration of time spent in the arms, was recorded during the 5-minute test.

#### Three-chamber assay

The three chamber assay measures animal sociability. A Plexiglas box (63 cm × 42 cm × 22 cm) was separated into 3 chambers (left, center and right) by removable dividing walls. Two identical inverted wire cup-like containers were placed in the left and right chambers and secured with a full water bottle on top of each to prevent the container from moving or being climbed. Two wild type male mice (Stranger 1 and 2) that were novel to (as well as age- and size-matched with) the test mice, were restrained individually in the containers for 5 minutes per day for 3–4 days prior to the experiment. On test days, a single wild type, *CBP*^+/ΔCH1^, or *CBP*^Δ*CH1/*Δ*CH1*^ mouse (Tester) was placed in the center chamber and allowed to freely explore all the three chambers for 10 minutes with both containers empty. After this habituation step, the sociability test was performed. The Tester was enclosed in the center chamber, and Stranger 1 was introduced into one of the containers. The dividing walls were removed and the time the Tester spent in each chamber and the duration of contact between the Tester and Stranger 1 or the empty container were recorded for 10 minutes. The discrimination index was calculated as [(Touch time _Stranger 1_ –Touch time _Container_) / total touch time] to reflect the degree of sociability. Next, the social recognition test was performed. Stranger 2 was placed into the empty container and the time the Tester spent in each chamber and the duration of contact between the Tester and Strangers 1 or 2 were recorded for 10 minutes. The discrimination index was calculated as [(Touch time _Stranger 2_ –Touch time _Stranger 1_) / total touch time] to indicate the degree of social recognition. After the set of tests for each Tester mouse, the chamber and containers were thoroughly cleaned to remove any residual scent. Animals showing no exploration were excluded. The location of empty containers and Strangers in the left and right chambers as well as the introduction order of Strangers 1 and 2 were systematically alternated.

#### Resident-intruder test

This assay measures aggressiveness. As described previously [27], 7–8 month-old male mice (resident) were singly housed for at least two weeks to establish dominance. During the experiment, a novel age- and size-matched wild type C57BL/6 ×129Sv F1 male mouse (intruder) was introduced into the cage. The latency to the first attack (boxing, chasing, biting, or dominant mounting) was recorded until a cutoff time of 10 minutes. Experiments were to be stopped if severe and intensive fighting occurred to avoid injuries to the mice, but no intensive fighting was observed during these tests.

#### Nest building skill assay

This assay tests the mouse’s home-cage activity linked to social function. In the test, mice were singly housed with normal bedding material and one folded Kimwipe. At 24, 48 and 72 hours, the manipulation of the Kimwipe and shape of the nest were scored on a 0–3 scale (0 = Kimwipe not noticeably touched; 1 = Kimwipe touched but no identifiable nest; 2 = an identifiable but flat nest; 3 = a (near) perfect nest with walls higher than the mouse body).

#### Wire hang test

The mouse was put on a wire cage lid and allowed to grasp it. The wire cage lid was then inverted and suspended 40 cm above the home cage. The latency to when the animal fell, with a test cutoff time of 120 seconds, was recorded to measure a mouse’s motor function. Three individual tests (with a 15-min interval between each test) were performed and the average latency was used.

#### Grip strength measurement

The grip strength of either the forelimbs alone or all four limbs was measured using the grip strength meter (Coulbourn) following the manufacturer’s instructions. Six independent measurements (with a 30-sec interval between each measurement) were taken and the average readings were used. Grip strength was measured as a control for the wire hang test.

#### Rotarod test

An accelerating rotarod apparatus (Ugo Basile) was used to test the motor function and motor learning of the mice. Up to 5 mice at a time were placed on the accelerating rotarod, which was linearly accelerated from 0 rpm to 40 rpm over the course of four minutes, then held at 40 rpm for the remainder of the test. The time to when each mouse fell off the rotarod within a cutoff time of 5 minutes was recorded. The mice were tested in four trials per day on two consecutive days, and allowed to rest for one hour between trials on the same day. The modified rotarod was described previously by Shahbazian *et al*. [28]. Briefly, the rod was covered with tape to minimize the grip and the mice were placed in either forward or backward direction before the rotation (0 to 40 rpm).

#### Object recognition assay

The object recognition assay provides a measure of the animals’ short- and long-term memory. Prior to training, each mouse was allowed to explore the testing chamber alone (48 cm × 26 cm × 20 cm) without the objects for 5 minutes on two consecutive days. During the 10-min training phase, the mouse was presented with two identical objects (Object A and A’). After a one-hour or 24-hour interval, a 10-min testing phase was carried out during which the mouse was re-introduced into the same chamber with one of the old objects (Object A’) replaced by an object with a novel color and shape (Object B). The time the mouse spent intentionally touching the object (investigating it, not brushing against it in passing) as well as the time the mouse was within 1 cm of the object and facing it were recorded as touch time. The discrimination index to measure the preference for the objects was calculated as [(Touch time Object A’ or B—Touch time Object A) / total touch time] to index the memory. Animals showing no exploration were excluded. The location, as well as the color and shape of the objects were systematically alternated [29].

### Electrophysiology

Three-month old mice were sacrificed and acute transverse hippocampal slices (400 μm) were prepared as previously described [30]. Briefly, mouse brains were quickly removed and placed in cold (4°C) dissecting artificial cerebrospinal fluid (ACSF) containing 125 mM choline-Cl, 2.5 mM KCl, 0.4 mM CaCl_2_, 6 mM MgCl_2_, 1.25 mM NaH_2_PO_4_, 26 mM NaHCO_3_, and 20 mM glucose (285–295 mOsm), under 95% O_2_/5% CO_2_. After dissection, slices were incubated for 1 hour in ACSF containing 125 mM NaCl, 2.5 mM KCl, 2 mM CaCl_2_, 2 mM MgCl_2_, 1.25 mM NaH_2_PO_4_, 26 mM NaHCO_3_, and 10 mM glucose (285–295 mOsm), under 95% O_2_/5% CO_2_ at room temperature and then transferred into submerged recording chambers and superfused (2–3 mL/min) with warm (30°C to 32°C) ACSF. Field recordings were performed using a setup with 8 submerged recording chambers (Campden Instruments, Lafayette, IN). Recordings in each chamber were performed independently. Field excitatory postsynaptic potentials (fEPSPs) from the CA1 stratum radiatum were recorded by using an extracellular glass pipette (3–5 MΩ) filled with ACSF. Schaffer collateral fibers in the stratum radiatum were stimulated with a bipolar tungsten electrode placed 200 to 300 mm away from the recording pipette. Stimulation intensities were chosen to produce an fEPSP with a 0.5 V/s slope. Long-term potentiation (LTP) was induced by 3 periods of 200-Hz tetanization delivered every 5 min. Each period of tetanization consisted of 10 trains of 200-Hz stimulation delivered at the same intensity for 200 ms (40 stimulations) every 5 s.

### Statistics

All results are presented as average ± SEM. The Student’s t-test was used to compare two groups with Gaussian distribution, and the Mann-Whitney test was used to compare two groups without Gaussian distribution. When comparing more than two groups, we used parametric ANOVA and Tukey’s post hoc analysis or non-parametric Kruskal-Wallis test and Dunnett’s post hoc analysis. Rotarod and electrophysiology were analyzed using ANOVA with repeated measures. The nest-building assay was analyzed using the Friedman Sum Rank test (for trial effect) and Kruskal-Wallis test (for group difference). All the statistics were performed using the Prism program (Graphpad).

## Results

### *CBP*^Δ*CH1/*Δ*CH1*^ mice have craniofacial anomalies

**Fig 1:**
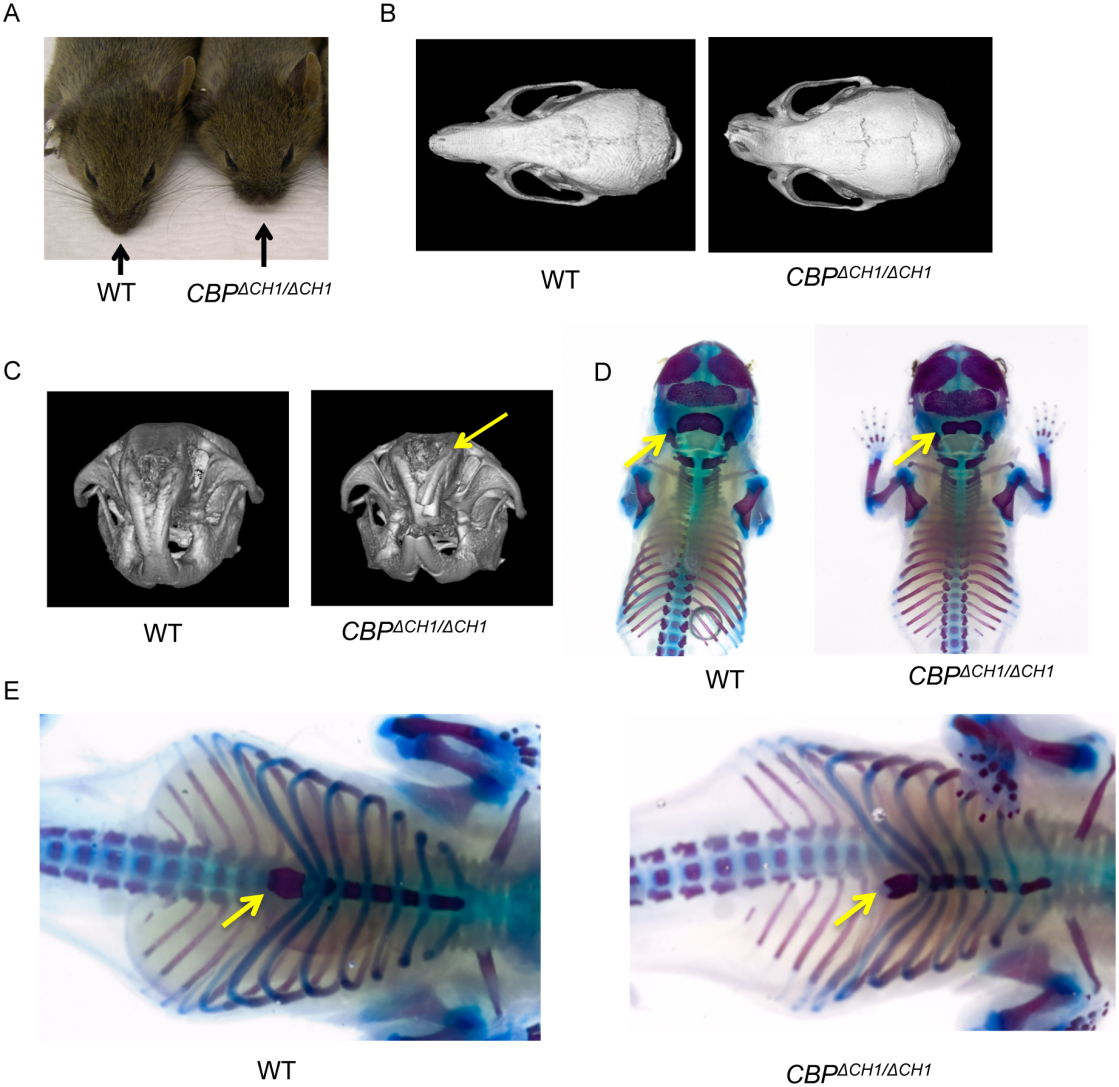
*CBP*^Δ*CH1/*Δ*CH1*^ mice display craniofacial anomalies. (A) *CBP*^Δ*CH1/*Δ*CH1*^ mice show shortened nasal bones with 100% penetrance. (B, C) MicroCT scans show that *CBP*^Δ*CH1/*Δ*CH1*^ mice have loss of lateral symmetry (B) and hyperdontia (C, see extra tooth indicated by yellow arrow) with partial penetrance. (D, E) Alcian Blue/Alizarin Red S staining of e18.5 embryos demonstrates additional developmental defects in the occipital bone of the skull (D) and bifurcation of the xyphoid process (E) in *CBP*^Δ*CH1/*Δ*CH1*^ embryos (both with 100% penetrance). Yellow arrows indicate occipital bone (D) and xyphoid process (E). *CBP*^Δ*CH1/*Δ*CH1*^ embryos all displayed decreased staining of cartilage with Alcian blue dye (compare wild type with *CBP*^ΔCH1ΔCH1^ embryos in D and E; different embryo pair represented in each panel).

Adult mice containing two alleles with the *CBP* CH1 deletion mutation (*CBP*^Δ*CH1/*Δ*CH1*^) are viable on a C57BL/6 X 129Sv F1 hybrid background, but not on pure (or non-F1 hybrid) C57BL/6 or 129Sv backgrounds [10,31]. Adult F1 hybrid *CBP*^Δ*CH1/*Δ*CH1*^ mice have normal life spans after weaning, but are smaller, have moderate craniofacial and skeletal anomalies (chiefly, blunt snouts, Fig 1), and are lean and insulin-sensitized [31].

### *CBP*^Δ*CH1/*Δ*CH1*^ mice display repetitive behaviors

**Fig 2:**
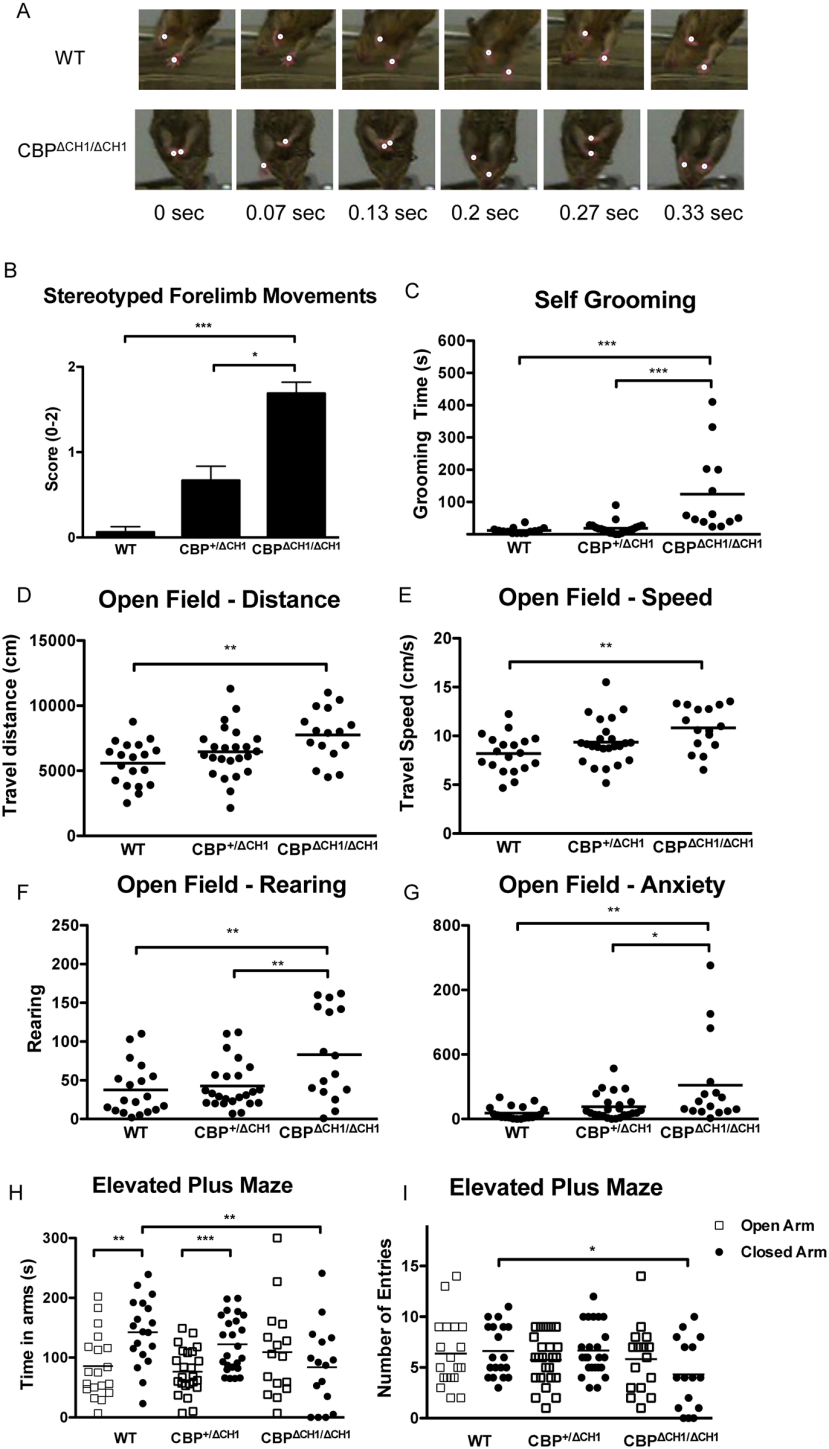
*CBP*^Δ*CH1/*Δ*CH1*^ mice show repetitive behaviors, hyperactivity, and less anxiety. (A,B) *CBP*^Δ*CH1/*Δ*CH1*^ mice display repetitive forelimb movements. White dots (A) indicate position of paws. Scores assigned in (B) represent the frequency of the repetitive movements. 0 = no forelimb repetitive movements (FRM); 1 = occasional FRM; 2 = continuous FRM. Mean ± SEM. N = 8 wild type (WT), 9 *CBP*^+/ΔCH1^, 8 *CBP*^Δ*CH1/*Δ*CH1*^. (C) *CBP*^Δ*CH1/*Δ*CH1*^ mice show significantly increased self-grooming time. N = 14 WT, 21 *CBP*^+/ΔCH1^, 13 *CBP*^Δ*CH1/*Δ*CH1*^. (D-F) *CBP*^Δ*CH1/*Δ*CH1*^ mice show increased travel distance (D), speed (E), and rearing (F) in a 30-min open field test. (G) *CBP*^Δ*CH1/*Δ*CH1*^ mice stay longer in the center of the open field arena. (H-I) *CBP*^Δ*CH1/*Δ*CH1*^ mice stay shorter in the closed arm, and enter less frequently the closed arm of an elevated plus maze. For (D-I), N = 19 WT, 24 *CBP*^+/ΔCH1^, 16 *CBP*^Δ*CH1/*Δ*CH1*^. For (B-I) Asterisks indicate the p value for either Dunnett’s (in the repetitive movement assay) or Tukey (in the other tests) post hoc analysis after one-way ANOVA (*: p<0.05; **: p<0.01; ***: p<0.001; ****: p< 0.0001). All the other pairings are not statistically different.

Strikingly, *CBP*^Δ*CH1/*Δ*CH1*^ mice exhibit stereotyped forelimb movements (Fig 2A and 2B; H (2) = 18.29, p = 0.0001; S1 Video (*CBP*^*+/+*^ mouse) and S2 Video (*CBP*^*ΔCH1/ΔCH1*^ mouse)), an autism-relevant repetitive behavior [2]. Moreover, *CBP*^Δ*CH1/*Δ*CH1*^ mice also spend increased time self-grooming, another autism-relevant repetitive behavior (Fig 2C and S1 Table; F (2, 45) = 12.59, p<0.0001). This grooming phenotype does not result from altered nociception because *CBP* CH1 mutant mice show a normal pain response in a hot plate assay (S2 Fig; F (2, 52) = 2.626, p = 0.082).

### *CBP*^Δ*CH1/*Δ*CH1*^ mice show hyperactivity and reduced anxiety

To determine whether *CBP* CH1 mutant mice display other autism-relevant behaviors, we performed open-field and elevated-plus-maze tests to measure locomotor activity and anxiety. In a 30 minute open-field test, *CBP*^Δ*CH1/*Δ*CH1*^ mice traveled farther than their wild type (WT) littermates (F (2, 56) = 5.754, p = 0.0053 for distance; F (2, 56) = 6.484, p = 0.0029 for speed), reared more frequently (F (2, 56) = 6.544, p = 0.0028), and spent more time in the central zone (F (2, 56) = 6.202, p = 0.0037) (Fig 2D–2F). In the elevated-plus-maze experiments, the mutants spent less time in the closed arm (F (2, 56) = 4.848, p = 0.0114) and entered the closed arm less frequently (F (2, 56) = 4.048, p = 0.0228) (Fig 2H and 2I). Interestingly, the total entry numbers were comparable among the three groups (WT 13.00±0.9673 vs. *CBP*^+/ΔCH1^ 12.42±0.7753 vs. *CBP*^Δ*CH1/*Δ*CH1*^10.13±1.363; F (2, 56) = 2.026, p = 0.1415). These results indicate that *CBP*^Δ*CH1/*Δ*CH1*^ mice display hyperactivity and less anxiety.

### *CBP*^Δ*CH1/*Δ*CH1*^ mice have impaired social interaction

**Fig 3:**
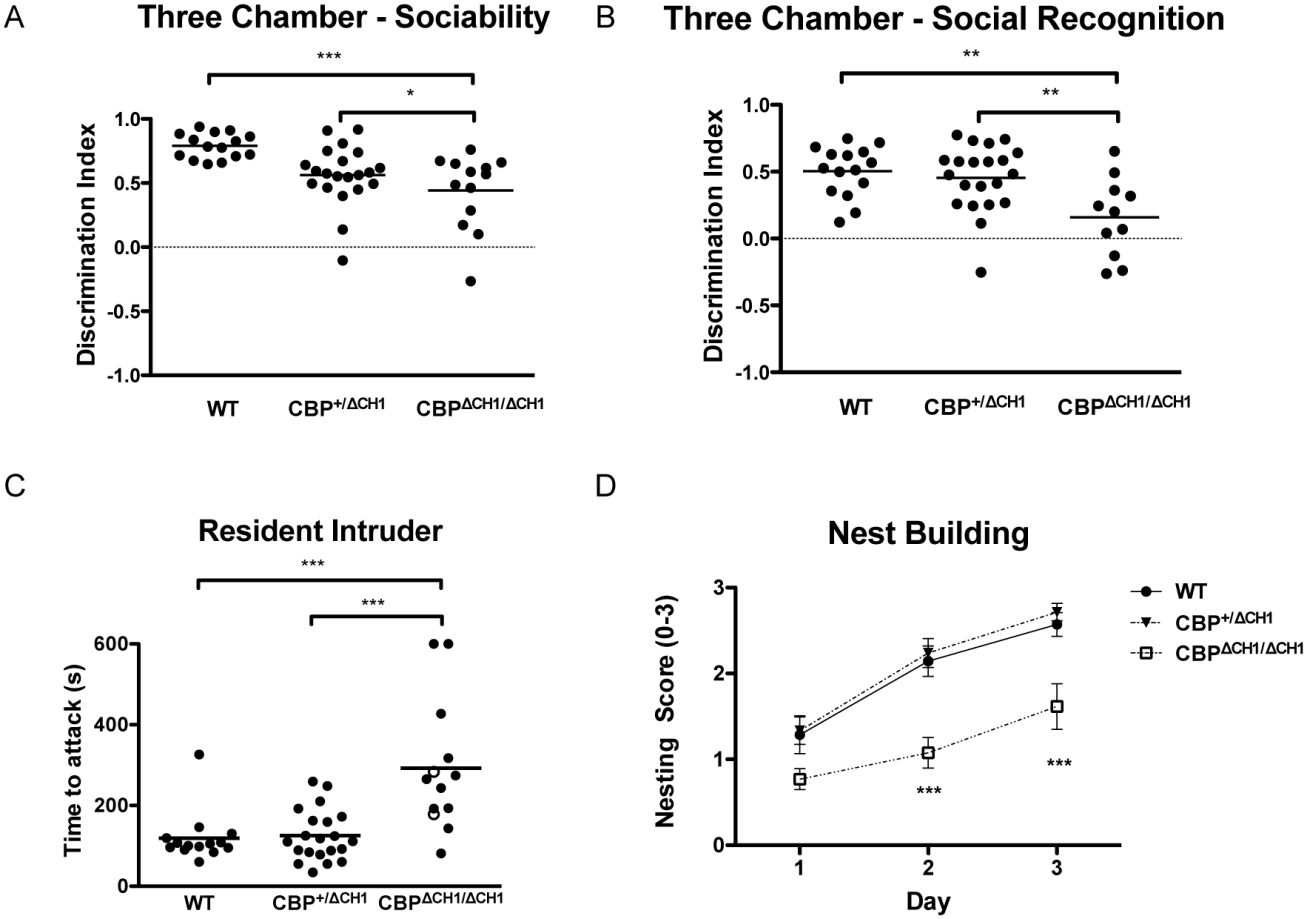
*CBP*^Δ*CH1/*Δ*CH1*^ mice show abnormal social behaviors. (A) *CBP*^Δ*CH1/*Δ*CH1*^ mice show reduced interest in an introduced animal in a three chamber-sociability assay. N = 15 wild type (WT), 21 *CBP*^+/ΔCH1^, 13 *CBP*^Δ*CH1/*Δ*CH1*^. (B) *CBP*^Δ*CH1/*Δ*CH1*^ mice display a decreased preference for the novel animal in a three chamber-social recognition assay. N = 15 wild type (WT), 21 *CBP*^+/ΔCH1^, 11 *CBP*^Δ*CH1/*Δ*CH1*^. (C) *CBP*^Δ*CH1/*Δ*CH1*^ mice are less aggressive. Open circles represent (unusual) attacks initiated by the intruder instead of the resident. (D) *CBP*^Δ*CH1/*Δ*CH1*^ mice have impaired nest-building skills. Nest building evaluated as follows: 0 = Kimwipe not notably touched; 1 = Kimwipe touched but no identifiable nest; 2 = an identifiable but flat nest; 3 = a (near) perfect nest with walls higher than the mouse body. For (C-D), N = 15 wild type (WT), 21 *CBP*^+/ΔCH1^, 13 *CBP*^Δ*CH1/*Δ*CH1*^. Asterisks indicate the p value for either Dunnett’s (in the nest building assay) or Tukey (in the other tests) post hoc analysis after ANOVA (*: p<0.05; **: p<0.01; ***: p<0.001; ****: p< 0.0001). All the other pairings are not statistically different.

We next asked if the *CBP* mutant mice have deficits in social interaction, which are also behavioral hallmarks of ASDs. We used a three-chamber assay to measure sociability and social recognition. Compared with their wild type littermates, the *CBP*^ΔCH1ΔCH1^ group spent significantly less time interacting with a mouse introduced into the chamber (F (2, 46) = 9.145, p = 0.0005; Fig 3A). They also showed a reduced preference for a novel versus a familiar mouse (F (2, 44) = 7.195, p = 0.0018; Fig 3B), suggesting that the CBP CH1 domain is required for normal sociability and social recognition. Moreover, in the resident-intruder paradigm that tests male-male aggressive behavior in social interaction, *CBP*^Δ*CH1/*Δ*CH1*^ mice showed much less aggression than wild type littermates (F (2, 45) = 13.83, p<0.0001; Fig 3C). In the nesting behavior assay, which has been proposed as a core test for autistic behaviors, *CBP*^Δ*CH1/*Δ*CH1*^ mice displayed poor nest building abilities (For day effect: WT, H (2) = 15.98, p = 0.0003389; *CBP*^+/ΔCH1^, H (2) = 29.278, p = 4.389e-7; *CBP*^Δ*CH1/*Δ*CH1*^, H (2) = 7.6158, p = 0.02219) (For group difference: Day1, H (2) = 6.020, p = 0.0493; Day2, H (2) = 17.14, p = 0.0002; Day3, H (2) = 14.44, p = 0.0007) (Fig 3D).

### *CBP*^Δ*CH1/*Δ*CH1*^ mice exhibit deficits in motor function and cognition

**Fig 4:**
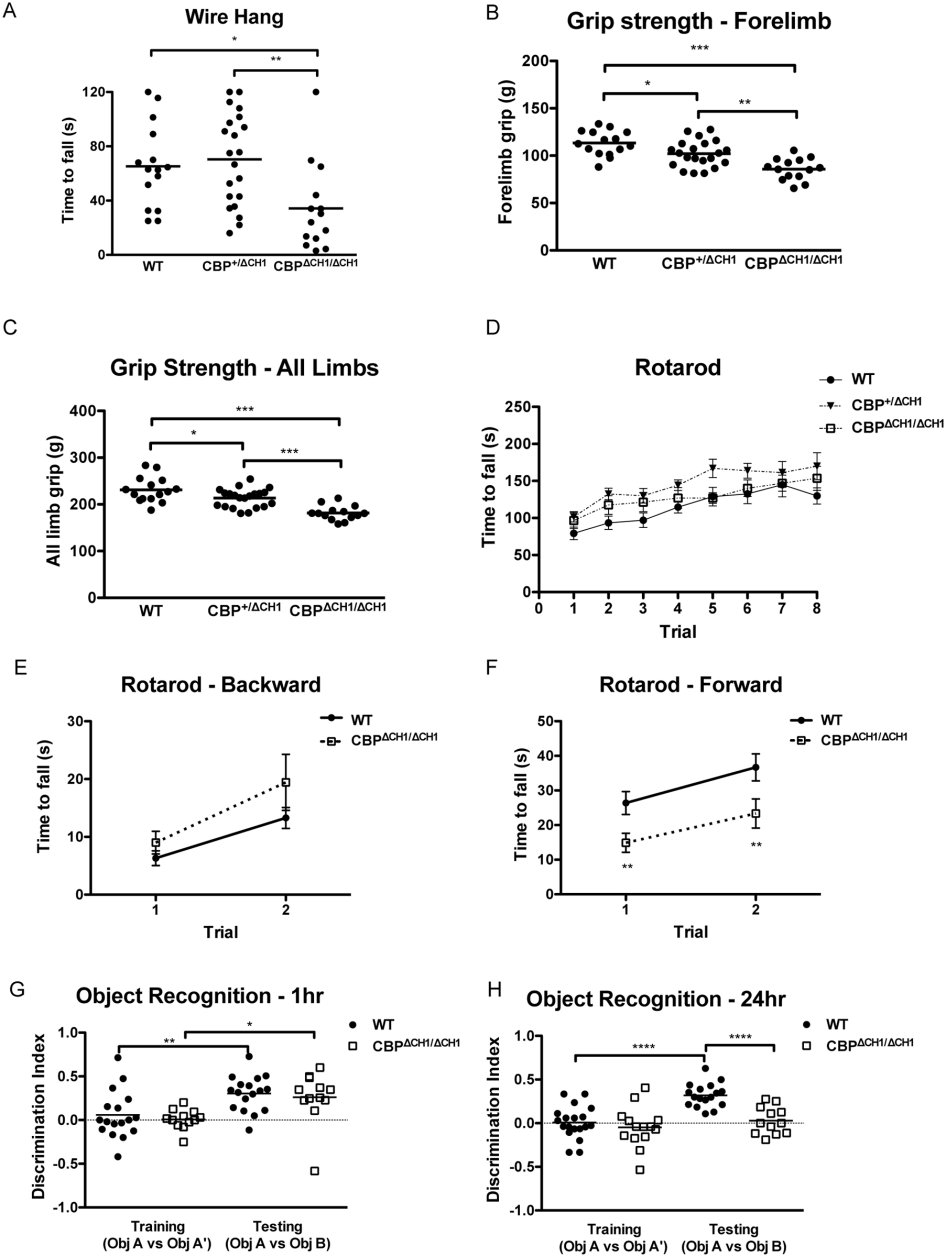
*CBP*^Δ*CH1/*Δ*CH1*^ mice display motor dysfunction and abnormal recognition memory. (A) *CBP*^Δ*CH1/*Δ*CH1*^ mice fall faster in a wire hang assay. N = 15 wild type (WT), 21 *CBP*^+/ΔCH1^, 14 *CBP*^Δ*CH1/*Δ*CH1*^. (B, C) *CBP*^Δ*CH1/*Δ*CH1*^ mice show significantly less grip strength for forelimbs only (B) or all four limbs (C). N = 15 WT, 21 *CBP*^+/ΔCH1^, 14 *CBP*^Δ*CH1/*Δ*CH1*^. (D) *CBP*^Δ*CH1/*Δ*CH1*^ mice perform normally in a classic rotarod assay. N = 10 WT, 12 *CBP*^+/ΔCH1^, 10 *CBP*^Δ*CH1/*Δ*CH1*^. (E,F) In a modified rotarod assay, in which the grips were eliminated from the rod surface, *CBP*^Δ*CH1/*Δ*CH1*^ mice perform normally when walking against the rod rotation (backward, E), but are impaired when walking with the rotation (forward, F). N = 42 WT, 27 *CBP*^Δ*CH1/*Δ*CH1*^. (G,H) In an object recognition test, *CBP*^Δ*CH1/*Δ*CH1*^ mice have intact short-term recognition memory but impaired long-term memory. N = 17 WT, 12 *CBP*^Δ*CH1/*Δ*CH1*^. Asterisks indicate the p value for the Student’s t-test (in the modified rotarod and the recognition memory test) or Tukey post hoc analysis after ANOVA in the other tests (*: p<0.05; **: p<0.01; ***: p<0.001; ****: p< 0.0001). All the other pairings are not statistically different.

Many patients with autism display motor dysfunctions and intellectual disabilities [4,32] that are also seen in RTS patients [22,23,33]. To determine if the CBP CH1 domain is involved in motor function, we performed a wire hang assay, and found that *CBP*^Δ*CH1/*Δ*CH1*^ mice fell from the wire more quickly than littermate controls (F (2, 47) = 5.679, p = 0.0062, Fig 4A). This may be explained by the reduced grip strength we measured in *CBP*^Δ*CH1/*Δ*CH1*^ mice (F (2, 47) = 16.34, p = <0.0001 for forelimb; F (2, 47) = 20.43, p = <0.0001for all limbs; Fig 4B and 4C). Although the *CBP*^Δ*CH1/*Δ*CH1*^ group showed a normal latency to fall from an accelerating rotarod (trials, F (7, 203) = 19.84, p<0.0001; genotypes, F (2, 29) = 3.17, p = 0.0569; trials X genotypes, F (14, 203) = 0.72, p = 0.7517; Fig 4D), when the apparatus was modified with tape to reduce surface friction, the mutants fell sooner when they walked with (forward, trials, F (1, 67) = 7.79, p = 0.0069; genotypes, F (1, 67) = 8.88, p = 0.0040; trials X genotypes, F (1, 67) = 0.07, p = 0.7893), but not against (backward, trials, F (1, 67) = 20.18, p<0.0001; genotypes, F (1, 67) = 2.22, p = 0.1410; trials X genotypes, F (1, 67) = 0.80, p = 0.3743), the direction of the rotating rod (Fig 4E and 4F). Together with the wire hang assay and grip strength measurements, the modified rotarod test results suggest impaired motor function in *CBP*^Δ*CH1/*Δ*CH1*^ mice. We next examined whether *CBP*^Δ*CH1/*Δ*CH1*^ mice display any cognitive deficits and found that they had impaired long-term recognition memory (t (27) = 5.339, p<0.0001) but intact short-term memory (t (27) = 0.4681, p = 0.6435) (Fig 4G and 4H), whereas WT and *CBP*^Δ*CH1/*Δ*CH1*^ mice interacted with the experimental objects for similar lengths of time (WT 52.83±5.91s vs. *CBP*^Δ*CH1/*Δ*CH1*^ 50.69±6.22s, p = 0.8091).

### *CBP*^Δ*CH1/*Δ*CH1*^ mice show abnormal synaptic plasticity

**Fig 5:**
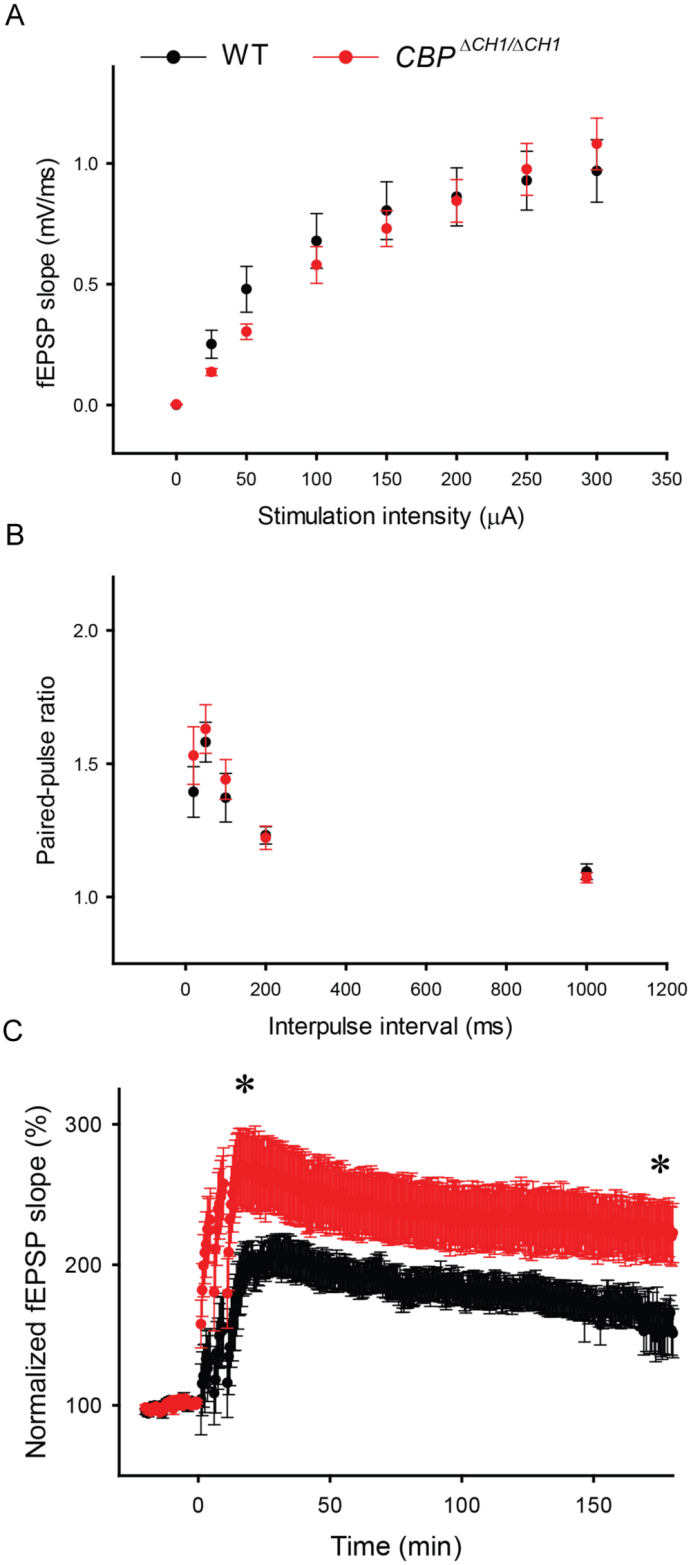
*CBP*^Δ*CH1/*Δ*CH1*^ mice showed increased hippocampal long-term potentiation (LTP). (A-C) Mean field excitatory postsynaptic potentials (fEPSPs) as a function of stimulation intensity (A), paired-pulse ratios as a function of interstimulus interval (B), and mean fEPSPs as a function of time before and after 200-Hz tetanus (applied at time 0) (C) measured at CA3-CA1 synapses in 2–3 slices per animal from WT (N = 4) and *CBP*^Δ*CH1/*Δ*CH1*^ (N = 5) mice. * p<0.05.

Altered synaptic plasticity has been reported in many ASD-relevant animal models, and it varies significantly between different models (for review, see [34]). Here we measured long-term potentiation (LTP) at excitatory synapses between CA3 and CA1 pyramidal neurons (CA3-CA1 synapses) in acute hippocampal slices from 3-month old *CBP*^Δ*CH1/*Δ*CH1*^ mice and their WT littermates. We found that the basal synaptic transmission and presynaptic function tested with paired-pulse facilitation are intact in slices from the mutant mice (Fig 5A and 5B), whereas the posttetanic potentiation (PTP) (p = 0.0383) and LTP (t (24) = 87.00, p = 0.018) were significantly enhanced (Fig 5C).

## Discussion

ASDs currently affect 1 out of 68 children [35]. Although a genetic component has already been identified in about 25% of ASDs [4], likely causative genes are still being identified. Here we showed that a deletion in the CBP CH1 domain leads to many autism-relevant phenotypes, including repetitive/stereotyped behaviors, aberrant sociability, reduced aggressiveness, hyperactivity, motor function deficits, and impaired recognition memory. These results suggest that CBP CH1 function is involved in pathways related to autism. This supposition is also supported by recent theoretical and mutational analyses of ASD patient families (S2 Table) [6,26].

Mutations in *CREBBP* lead to Rubinstein-Taybi Syndrome (RTS), which is characterized by intellectual disability (ID) but not autism *per se* [14,36]. However, some evidence suggests that ID and ASD share similar cellular and molecular mechanisms (reviewed in [37]). Indeed, autistic behavior has been reported in some RTS patients, and is more common in patients bearing large *CREBBP* deletions [23]. In addition, many genome-wide studies including gene association analysis and whole exome sequencing have implicated *CREBBP* as an autism candidate gene, or interaction hub [6,26,38,39]. Moreover, CBP mRNA and protein levels are reportedly decreased in the frontal gyrus of patients with autism [40]. Given this, the question remains why only a portion of RTS patients have autism-like symptoms. One possibility is that overall genetic context (i.e. genetic modifiers) affects which symptoms are displayed in human RTS patients. Supporting the role of genetic modifiers in determining the severity of symptoms produced by CBP mutations, we find that *CBP* CH1 homozygous mutant mice can survive as adults only on a F1 hybrid genetic background [10,31]. Alternatively, pleiotropic phenotypes (e.g. RTS, death) caused by severe mutations in CBP or p300 in humans and mice may mask ASD-relevant symptoms.

Several CBP mutant mice have been generated as RTS models [16,18,19,29,41], and they all present certain RTS-like symptoms (S1 Table). They all showed similar phenotypes including cognition deficits. Because long-term memory formation depends on gene expression, CBP, as a transcriptional coactivator, regulates many important genes required for memory formation (e.g. *cFos*, *Arc*, *Bdnf*) [29]. Consistently, we found *CBP*^Δ*CH1/*Δ*CH1*^ mice had intact short-term memory and impaired long-term memory. However, some phenotypes are not consistent between RTS models. For example, one CBP truncation model [18] showed hypoactivity, whereas *CBP*^Δ*CH1/*Δ*CH1*^ mice display hyperactivity and other models showed normal locomotor activity (S1 Table). Although the correlation between activity and RTS is still unclear, hyperactivity is a frequent comorbidity observed in ASD patients and in several ASD-relevant mouse models [2,4,33,42,43]. Notably, *CBP*^Δ*CH1/*Δ*CH1*^ mice showed normal activity during the social behavior tests and cognition tests. On one hand, this may indicate that the reduced interaction with animal or objects is due to impaired sociability or memory but not altered activity. On the other hand, the differing activity observations between the open field test and other tests remain elusive. This could result from the different environment (novel vs. habituated) or the test context (other animal/objects involved).

No specific autism-relevant phenotypes in RTS mouse models have been reported previously. Thus, the key CBP functional defects that lead to RTS-like symptoms, especially with respect to autism-like symptoms, remain unclear. Contributing to this uncertainty are the over 400 different proteins reported to interact with CBP and p300, many of which represent components of different transcriptional pathways [9]. Transcriptional activators (e.g. CREB and HIF) recruit CBP through interaction domains (e.g. KIX or CH1, respectively) and the recruited CBP then acetylates histones (and other proteins) via the HAT domain, or recruits additional cofactors, which then contribute to transcriptional modulation. Inactivation of the whole CBP protein or its HAT domain potentially impacts many different and unrelated transcriptional pathways. Mutation of the protein-interaction domains themselves (e.g. KIX, CH1), however, can dissect certain aspects of CBP function. For example, CBP KIX mutant mice have cognition deficits but normal craniofacial development [29], whereas CBP CH1 mutant mice have craniofacial anomalies and autism-relevant phenotypes. Interestingly, EPAS1 (HIF2), a transcription factor important in the hypoxic response and that interacts with the CH1 domain, was recently identified as a novel autism risk gene [44]. Previous research has shown that the ΔCH1 mutation produces altered gene expression in response to hypoxia [10], and may represent a mechanism by which CBP/p300 modulate autism-relevant gene expression.

Intriguingly, *CBP* CH1 mutant mice share similar phenotypes with *Mecp2* mutant mice (S1 Table). MECP2 (methyl CpG binding protein 2) is a methylated DNA binding factor, and mutations in *MECP2* cause Rett syndrome [45]. Mice with different mutations in *Mecp2* have been generated as Rett-relevant models, and the type of *Mecp2* mutation produces somewhat different effects on mouse social interactions and repetitive behavior [4]. *CBP*^Δ*CH1/*Δ*CH1*^ mice exhibit repetitive forelimb movements that are very similar to those previously reported for mice expressing MECP2 truncated at residue 308 [28] and mice carrying an isoform-ablating *Mecp2* exon 1 (e1) mutation [46]. The presence of involuntary hand movements is a diagnostic feature of Rett syndrome patients [47]; however, repetitive forelimb movement is not a commonly reported autism-relevant behavior in mice (source: Mouse Genome Informatics database). Notably, repetitive hand clapping or flapping is also reported in RTS patients [22]. These unique forelimb movements, as well as many other shared phenotypes, suggest that CBP and MECP2 converge on a common molecular or cellular mechanism that may explain aspects of RTS and Rett syndrome. One logical hypothesis is that converging neuronal functions are dependent on interaction between MECP2 and the CH1 domain of CBP. Previous reports also suggest that MECP2 interacts with CREB [48], the archetype CBP binding partner, and that the CBP paralog, p300, can acetylate MECP2 [49]. An interaction between CBP and MECP2 might be physical (e.g. direct binding or via an adaptor protein), spatial (binding in the same genomic region, such as a promoter), or temporal (acting at different times during a process such as transcription). MECP2 and CBP CH1 may also converge via distinct developmental pathways that affect a particular cell type.

Recent studies suggest that abnormal synaptic homeostasis may be a key cellular mechanism of autistic behaviors (for reviews, see [50,51]). We investigated the synaptic plasticity of *CBP* CH1 mutant mice and found two interesting phenomena. First, mutation of the *CBP* CH1 domain has no effect on the basal synaptic transmission, suggesting the mutant mice developed normal and functional synapses. Second, the synaptic plasticity of *CBP* CH1 mutant mice was altered and the hippocampal LTP showed enhancement. Enhanced LTP has been reported in *Mecp2* transgenic mice (another Rett model) [52] and many other ASD models [43,53,54], indicating that enhanced LTP is also associated with autistic features. Furthermore, it has been widely accepted that abnormal strengthening of synapses also has deleterious effects on learning and memory [30,55–57], which may explain the impaired memory seen in *CBP*^Δ*CH1/*Δ*CH1*^ mice. The effect of CBP mutation on synaptic plasticity may also vary according to genetic background, age, and induction protocol. For instance, *in utero* exposure to valproic acid, a histone-deacetylase inhibitor, results in autism-relevant behaviors in rats, and modifies NMDA receptor synaptic expression as well as synaptic plasticity in an age-dependent manner (increasing in youth and decreasing in adulthood) [58]. This suggests that acetylation regulates synaptic function differently depending on the developmental stage. It has also been noted that overexpression of truncated CBP in postnatal forebrain neurons affects only certain forms of LTP [41].

Here we demonstrated that an intact CH1 domain in CBP is important for normal social behavior, motor function, and cognition, suggesting that reduced CH1 domain function is one mechanism that contributes to RTS. *CBP* CH1-deficient mice show behaviors reminiscent of mouse models for RTS, Rett syndrome, and ASDs, implicating the CBP CH1 domain in a converging pathway, and providing insight for future mechanistic studies of several neurological diseases.

## Supporting Information

S1 Fig CBP protein domain scheme adapted from Dyson and Wright [59]. Principle CBP domains include: nuclear receptor interaction domain (NRID), the Cys/His-rich region 1 (CH1 or TAZ1), the CREB-binding domain (KIX), bromodomain (Br), plant homeodomain (PHD), histone acetyltransferase domain (HAT), zinc-binding domain near the dystrophin WW domain (ZZ), the Cys/His-rich region 3 (CH3 or TAZ2), and the nuclear coactivator binding domain (NCBD or iBID).
(PDF)

S2 Fig *CBP*^Δ*CH1/*Δ*CH1*^ mice have comparable nociception with their WT littermates. N = 24 WT, 19 *CBP*^+/ΔCH1^, 12 *CBP*^Δ*CH1/*Δ*CH1*^.
(PDF)

S1 Table Phenotype comparison of CBP and Mecp2 mutant mouse models. N.R., not reported; N.O.P., no obvious phenotype (Zheng et al, unpublished data).
(PDF)

S2 Table Bioinformatic analysis of *CREBBP* and *EP300* de novo ASD mutations identified by Iossifov *et al*. 2014 [26]. (PDF)

S1 Video No forelimb rubbing observed in wild type control mice. (AVI)

S2 Video Forelimb rubbing phenotype observed in *CBP*^Δ*CH1/*Δ*CH1*^ mice. (AVI)
