# Supplementary figures and images for: Mutation of the CH1 Domain in the Histone Acetyltransferase CREBBP Results in Autism-Relevant Behaviors in Mice
Source: PLoS One. 2016 Jan 5;11(1):e0146366. doi: 10.1371/journal.pone.0146366 (PMC4701386; doi:10.1371/journal.pone.0146366)

Supplemental Figure 1

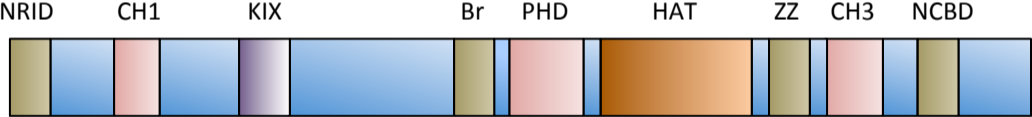

Supplement: S1 Fig — Principle CBP domains include: nuclear receptor interaction domain (NRID), the Cys/His-rich region 1 (CH1 or TAZ1), the CREB-binding domain (KIX), bromodomain (Br), plant homeodomain (PHD), histone acetyltransferase domain (HAT), zinc-binding domain near the dystrophin WW domain (ZZ), the Cys/His-rich region 3 (CH3 or TAZ2), and the nuclear coactivator binding domain (NCBD or iBID). (PDF) [file pone.0146366.s001.pdf]

## S2 Figure

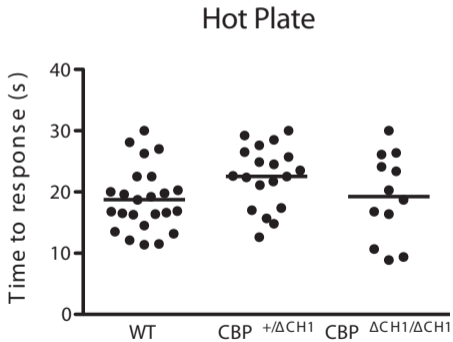

Supplement: S2 Fig — N = 24 WT, 19 CBP+/ΔCH1, 12 CBPΔCH1/ΔCH1. (PDF) [file pone.0146366.s002.pdf]
