# Supplementary material for: Mutation of the CH1 Domain in the Histone Acetyltransferase CREBBP Results in Autism-Relevant Behaviors in Mice
Source: PLoS One. 2016 Jan 5;11(1):e0146366. doi: 10.1371/journal.pone.0146366 (PMC4701386; doi:10.1371/journal.pone.0146366)
Supplement: S1 Table — N.R., not reported; N.O.P., no obvious phenotype (Zheng et al, unpublished data). (PDF) [file pone.0146366.s003.pdf]

S1 Table. Phenotype comparison of CBP and Mecp2 mutant mouse models.

	$CBP^{\Delta CH1/\Delta CH1}$	CBP+/-	CBP Trunc	CBPKIX	CBPcKO	CBPΔ1	$Mecp2^{-/Y}$	$Mecp2^{308/Y}$	$Mecp2\text{-}e1^{-/Y}$
**Mutations**	Deletion in CH1 domain in CBP	Deletion from exon 2[a]	Truncation at amino acid 1084[b]	Triple point mutations in CBP KIX domain[c]	Deletion of CBP in forebrain[d]	O/E truncated CBP in forebrain[h]	Deletion of exon 3 or exon 3 & 4[e]	Truncation at amino acid 308[f]	Mutation in exon 1 (e1 form deletion)[g]
**Stereotypies**	Forepaw rubbing; Self grooming	N.R.	N.R.	N.O.P.	N.R.	N.R.	Self grooming	Forepaw rubbing; Self grooming	Forepaw rubbing; Self grooming
**Spasticity**	N.O.P.	N.R.	N.R.	N.O.P.	N.R.	N.R.	Hindlimb clasping	Hindlimb clasping	Hindlimb clasping
**Social behavior abnormalities**	Yes	N.R.	N.R.	N.R.	N.R.	N.R.	Yes	Yes	Yes
**Motor dysfunction**	Yes	Yes	N.R.	Yes	No	No	Yes	Yes	Yes
**Locomotor activity**	Hyperactivity	Normal	Hypoactivity	Normal	Normal	Normal	Hypoactivity	Hypoactivity	Normal
**Anxiety**	Decreased	Normal	Normal	Normal	Normal	Normal	Decreased	Increased	Decreased
**Learning and memory deficits**	Yes	Yes	Yes	Yes	Yes	Yes	Yes	Yes	N.R.
**LTP**	Enhanced	Reduced	Reduced	Reduced	N.R.	Reduced	Reduced	Reduced	N.R.

[a] Tanaka et al., 1997, Alarcon et al., 2004
[b] Oike et al., 1999
[c] Wood et al., 2006
[d] Valor et al., 2011
[e] Wood et al., 2005
[f] Chen et al., 2001, Guy et al., 2001
[g] Shahbazian et al., 2002, Moretti et al., 2006
[h] Yasui et al., 2014

Supplemental Reference:

Alarcon JM, Malleret G, Touzani K, Vronskaya S, Ishii S, Kandel ER, Barco A (2004) Chromatin acetylation, memory, and LTP are impaired in CBP+/- mice: a model for the cognitive deficit in Rubinstein-Taybi syndrome and its amelioration. Neuron 42:947-959.

Chen RZ, Akbarian S, Tudor M, Jaenisch R (2001) Deficiency of methyl-CpG binding protein-2 in CNS neurons results in a Rett-like phenotype in mice. Nat Genet 27:327-331.

Guy J, Hendrich B, Holmes M, Martin JE, Bird A (2001) A mouse Mecp2-null mutation causes neurological symptoms that mimic Rett syndrome. Nat Genet 27:322-326.

Moretti P, Levenson JM, Battaglia F, Atkinson R, Teague R, Antalffy B, Armstrong D, Arancio O, Sweatt JD, Zoghbi HY (2006) Learning and memory and synaptic plasticity are impaired in a mouse model of Rett syndrome. J Neurosci 26:319-327.

Oike Y, Hata A, Mamiya T, Kaname T, Noda Y, Suzuki M, Yasue H, Nabeshima T, Araki K, Yamamura K (1999) Truncated CBP protein leads to classical Rubinstein-Taybi syndrome phenotypes in mice: implications for a dominantnegative mechanism. Hum Mol Genet 8:387-396.

Shahbazian M, Young J, Yuva-Paylor L, Spencer C, Antalffy B, Noebels J, Armstrong D, Paylor R, Zoghbi H (2002) Mice with truncated MeCP2 recapitulate many Rett syndrome features and display hyperacetylation of histone H3. Neuron 35:243-254.

Tanaka Y, Naruse I, Maekawa T, Masuya H, Shiroishi T, Ishii S (1997) Abnormal skeletal patterning in embryos lacking a single Cbp allele: a partial similarity with Rubinstein-Taybi syndrome. Proc Natl Acad Sci U S A 94:10215-10220.

Valor LM, Pulopulos MM, Jimenez-Minchan M, Olivares R, Lutz B, Barco A (2011) Ablation of CBP in forebrain principal neurons causes modest memory and transcriptional defects and a dramatic reduction of histone acetylation but does not affect cell viability. J Neurosci 31:1652-1663.

Wood MA, Attner MA, Oliveira AM, Brindle PK, Abel T (2006) A transcription factorbinding domain of the coactivator CBP is essential for long-term memory and the expression of specific target genes. Learn Mem 13:609-617.

Wood MA, Kaplan MP, Park A, Blanchard EJ, Oliveira AM, Lombardi TL, Abel T (2005) Transgenic mice expressing a truncated form of CREB-binding protein (CBP) exhibit deficits in hippocampal synaptic plasticity and memory storage. Learning & memory 12:111-119.
