# Supplementary material for: Mutation of the CH1 Domain in the Histone Acetyltransferase CREBBP Results in Autism-Relevant Behaviors in Mice
Source: PLoS One. 2016 Jan 5;11(1):e0146366. doi: 10.1371/journal.pone.0146366 (PMC4701386; doi:10.1371/journal.pone.0146366)
Supplement: S2 Table — (PDF) [file pone.0146366.s004.pdf]

S2 Table. Bioinformatic analysis of *CREBBP* and *EP300* de novo ASD mutations identified by Iossifov *et al.* .

Gene	Human Chrom	Variant base	Effect	Residue affected	Mutation	Conserved residue?	Domain
CREBBP	16:3842074	C->T	missense	Arg413	R413Q	highly (vertebrate & invertebrate)	CH1
CREBBP	16:3786149	T->C	missense	Tyr1539	Y1539C	highly (vertebrate & invertebrate)	HAT
CREBBP	16:3786060	T->C	missense	Thr1569	T1569A	partly (vertebrate)	HAT
CREBBP	16:3779789	G->C	silent	Gly1753	G1753G	NA	interdomain
EP300	22:41527522	G->A	silent	Pro481	P481P	NA	interdomain
EP300	22:41572350	C->T	missense	Arg1627	R1627W	highly (vertebrate & invertebrate)	HAT
EP300	22:41573317	A->C	missense	Thr1868	T1868P	partly (vertebrate p300)	interdomain
